# The State in Which Oxygen Exists in the Blood

**Published:** 1852-09

**Authors:** 


### CHEMISTRY.

*The State in which Oxygen exists in the Blood.*—Liebig, in the *Chemisch-Pharmaceutisches Central-Blatt,* Dec. 1851, has published a memoir on this subject, in which he combats the opinion that oxygen is not chemically combined with, but is simply absorbed by the blood; a belief grounded on the fact of the expulsion of the absorbed oxygen by a current of carbonic acid passing through the liquid. The author denies the correctness of this inference on the following grounds: 1000 volumes of water, according to Gay-Lussac, when saturated with atmospheric air, absorb 9·25 volumes of oxygen, and 18·5 volumes of nitrogen; whilst, according to Magnus, 1000 volumes of blood absorb from 100 to 130 volumes of oxygen, and 17 to 33 volumes of nitrogen; therefore blood absorbs from eleven to fourteen times more oxygen than water does, which absorption must necessarily depend upon the existence in the blood of some particular substance, possessing a far stronger affinity for oxygen than water possesses. It does not follow because the the affinity of this principle in the blood for oxygen is but weak, that no chemical combination exists between them. One per cent. of phosphate of soda in water, doubles the absorbent power of the water for carbonic acid gas; sulphate of iron in solution dissolves forty times more than a similar bulk of pure water can; and both these solutions again part with their absorbed gases, when placed *in vacuo*, or by agitation of the former with air, the latter with carbonic acid; so that the conclusion is obvious, that saline solutions possess (*may possess*) greater absorbent powers than pure water—a conclusion which applies to the blood.

The absorbent power of a liquid for gases, depends either upon the pressure maintained, or the affinity of that liquid, or of one or more of the constituents of that liquid, for a given gas. Were the oxygen of the blood simply absorbed, it must take up from the air, which itself contains but one-fifth of oxygen, twelve per cent. of this gas; double this amount under twice the pressure; treble under thrice this amount of pressure; and five times the quantity if shaken with pure oxygen. It has not yet been proved that the absorbent power of blood varies with the amount of atmospheric pressure. Regnault and Reiset have demonstrated that animal life can be maintained in an atmosphere abounding with oxygen; and it is well known that in the neighborhood of Lake Titicaca, at an elevation of 12,600 feet above the sea level, 15,000 persons, and that at Potosi, an elevation of 12,600 feet, 30,000 persons live and breathe every whit as well as the inhabitants of the coast; facts which militate against the supposition that oxygen is merely *absorbed* by the blood; for as absorption is dependent upon pressure, the amount absorbed at different elevations would be dissimilar, which must necessarily exert considerable influence on the vital action in the mountaineers, or on the dwellers on the sea-shore—a conclusion utterly opposed to observation.—*London Journal of Medicine.*

